# Initial Presentation of Acute Lymphoblastic Leukemia with Osteoporosis and Multiple Spontaneous Bone Fractures

**Published:** 2011-01-01

**Authors:** N Cohan, S Sarikhani, S Moslemi, M Karimi

**Affiliations:** 1 Hematology Research Center, Nemazee Hospital, Shiraz University of Medical Sciences, Shiraz, Iran; 2 College of Medicine, Shiraz University of Medical Sciences, Shiraz, Iran

**Keywords:** Acute lymphoblastic leukemia, Osteoporosis, Bone fracture, Iran

## Abstract

Acute lymphoblastic leukemia (ALL) is the most common childhood malignancy. Skeletal abnormalities have been described in association with ALL including osteoprosis and bone fractures. Different factors including the disease itself or soluble products of malignant cells and treatment agents like cytotoxic drugs, methotroxate, corticosteroids and radiotherapy may be responsible for defective bone homeostasis in these patients. Orthopedic conditions and pain may be the first manifestation of acute leukemia and it is important for physicians to recognize the skeletal manifestation of acute childhood leukemia because of a delay in diagnosis has adverse effect on survival. We present a child with ALL that refer with multi bone fractures as a first manifestation of the disease.

## Introduction

An Acute lymphoblastic leukemia (ALL) is the most common childhood malignancy represents about 35% of all childhood malignancies. The first symptoms of ALL are usually non specific. Although ALL is primarily a disease of bone marrow and peripheral blood, any other organ may be infiltrated by leukemic cells.[1] Skeletal abnormalities have been described in association with ALL, including osteoporosis, periostal reaction, reactive sclerosis, lytic defects and vertebral compression fractures. Children with ALL often have bone pain and disturbances of gait. Reduced bone turn over has been reported at diagnosis and during the treatment of ALL.[2][3] Osteoporosis is currently receiving an increasing attention as an important late effect in survivors of childhood cancer and its treatment because of their quality of life and its negative effect on the survivors ability to perform developmentally appropriate activities.[4] In southern Iran, the crude incidence rate and ASR of ALL were 2.54 and 2.18 and these figures for the females were 1.81 and 1.5 reported among the first 10 prevalent cancer in Fars Province, southern Iran.[5] We report an ALL patient who referred to our ward with complain of osteoporosis, bone pain and multi bone fractures complain firstly.

## Case Report

**Fig. 1:**
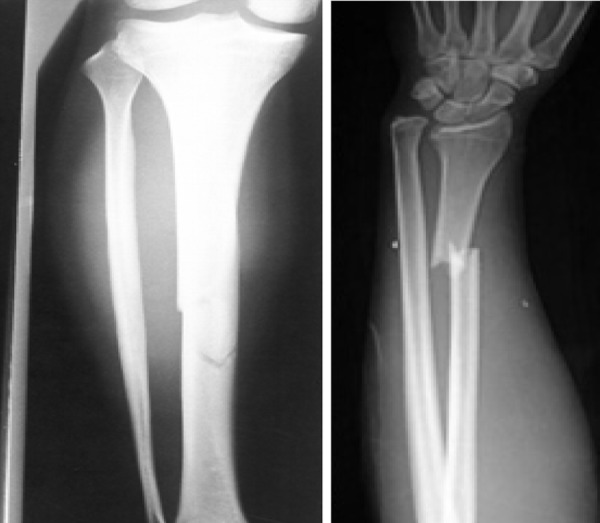
Bone fractures in patient with ALL.

A 4-year-old boy, suffering from recently developed multiple spontaneous bone fractures referred to Nemazee Hospital, Pediatric Center, for a precise diagnosis of the causes and management. The patient had developed a spontaneous right tibial fracture, proceeding by right femoral, nasal and finger fractures in a 1.5-month period (Figure 1). In addition, a generalized osteoporosis was obvious in X-rays of ribs, humerous and vertebral bones, also the X-rays revealed tibial fracture (in the right side) and femoral bone fracture in the same side, in addition right iliac fracture was observed.

Also the patient had a history of anorexia, nausea, pallor, disseminated bone pain, abdominal distention and severe generalized edema since 7 days prior to this admission. Pallor, generalized petechial lesions all over his body and face, huge peripheral lymphadenopathies (each one 4*3 cm ), a mild splenomegally, and a grade 4/6 systolic heart murmur with radiation to the left side of his chest wall were the remarkable points in physical examination. Growth percentiles of the patient were all in normal ranges.

Complete blood count (CBC) showed, white blood cells (WBC) 1.8×10^3^/µl with 80% lymphocyte and 20% neutrophil, red blood cells (RBC) 2.7×10^6^/ µl, platelet count (Plt) 90×10^3^/µl, hemoglobin (Hb) 6.4 gr/dl and erythrocyte sedimentation rate (ESR) 146 mm/hr. Other abnormal tests were urine calcium level 18.1 mg/dl, Ca 9.3 mg/dl, P 4.1 mg/dl and Alk-ph 1500 IU/L.

Bone marrow aspiration was done and showed a hypocellular marrow containing more than 90% lymphoblasts and immunophenotyping showed B-cell ALL.

B-cell ALL induction chemotherapy was started as follows: Prednisolone, vincristine, asparginase, daunorubicin, methotroxate and cytosin arabnoside. The patient developed complete remission. Then it has been a maintenance therapy as well as two courses of consolidation therapy. According to type of fractures (green stick) any workups for management of fractures not done for him. In follow up X-rays, callus formation was seen.

## Discussion

Decreased bone mineral density (BMD) which can lead to fractures, deformity and pain has been recognized in children with ALL.[4] Acute leukemia of childhood may present with various manifestations that mimic orthopedic conditions. Skeletal manifestation in children with ALL are reported within a wide variety and distribution with incidence rate ranging from 10% to 40%.[6]

Skeletal radiographic changes that can occur in a child with acute leukemia include diffuse osteopnia, metaphysial bands, periosteal new bone formation, geographic osteolysis, osteosclerosis, mixed osteolysis-sclerosis and permeative destruction.[7]

Hesseling et al. have reported initial radiographic findings, most frequently as slight metaphysial transverse lucent bands with or without diffuse demineralization, and bone changes such as periosteal reaction with or without intramedullary osteolytic mottling, in 9% of patients with leukemia.[8]

Our case was a patient with ALL that referred with multi bone fractures at the time of presentation. The pathogenesis of decreased BMD in childhood ALL is multifactor including soluble products of malignant cells,[2] invasion of bone by leukemia cells, corticosteroid and methoteroxate treatment and radiation.[4][9]

In conclusion, orthopedic conditions including multiple bone fractures may be the first manifestation of acute leukemia and it is important for physicians to recognize the skeletal manifestations of acute leukemia in childhood because of a delay in diagnosis has an adverse effect on survival.
